# Think Aloud Testing of a Smartphone App for Lifestyle Change Among Persons at Risk of Type 2 Diabetes: Usability Study

**DOI:** 10.2196/48950

**Published:** 2023-11-15

**Authors:** Pernille Lunde, Gyri Skoglund, Cecilie Fromholt Olsen, Gunvor Hilde, Way Kiat Bong, Birgitta Blakstad Nilsson

**Affiliations:** 1 Department of Rehabilitation Science and Health Technology Faculty of Health Sciences Oslo Metropolitan University Oslo Norway; 2 Department of Computer Science Faculty of Technology, Art and Design Oslo Metropolitan University Oslo Norway; 3 Section for Physiotherapy Division of Medicine Oslo University Hospital Oslo Norway

**Keywords:** mHealth, mobile phone app, smartphone, lifestyle, usability, diabetes, diabetic, mobile health, smartphone, app, apps, application, applications, think-aloud, think aloud, user experience, mobile phone

## Abstract

**Background:**

Type 2 diabetes mellitus (DM2) is a leading cause of morbidity and mortality worldwide and is considered a global epidemic. Despite the growing evidence on the effectiveness of mobile health interventions in the management of DM2, the evidence on the effect of mobile health interventions in prevention of DM2 is sparse. Therefore, we have developed an app aiming to promote initiation of behavioral change and adherence to healthy behavior. Before commencing a small-scale randomized controlled trial to assess the feasibility of using an app for initiation and adherence of healthy behavior in people at risk of DM2, testing the usability of the app in the target population is warranted.

**Objective:**

The aim of this study was to assess the usability of an app among people at risk of DM2.

**Methods:**

A qualitative study with the use of a think aloud (TA) procedure was conducted from April to November 2022. The TA procedure consisted of 10 problem-solving tasks and a semistructured interview which was carried out after the tasks. These interviews served to gain more in-depth knowledge of the users experience of the problem-solving tasks. The TA-sessions and the postactivity interviews were recorded and transcribed verbatim, and the data were coded and analyzed following the principles of thematic analysis.

**Results:**

In total, 7 people at risk of DM2 with a median age of 66 (range 41-75) years participated in this study. The analysis resulted in the following themes: (1) user interface design; and (2) suggestions for improvements of the functionality of the app.

**Conclusions:**

Overall, the participants were satisfied with the usability of the app. Through the TA-sessions, real time perspective on the appeal, relevance, and utility of the app were gained. Only minor changes to the functionality of the prototype app were reported as necessary to improve the usability of the app. Points of guidance from the participants in this study have been adopted and incorporated into the final design of the app now being assessed for feasibility in a small-scale randomized controlled trial.

## Introduction

Type 2 diabetes mellitus (DM2) is a leading cause of morbidity and mortality worldwide and is considered a global epidemic [1,2]. Urgent public health and clinical preventive measures are needed [3]. Behavior change is considered as a cornerstone in the prevention of DM2 [4,5], and mobile health (mHealth) interventions have been proposed to meet the challenges related to initiation and adherence to healthy behavior [6,7]. mHealth, defined as the medical and public health practice supported by mobile devices [8] includes, among other things, smartphone apps. Apps have the potential to deliver a diversity of behavioral interventions which in turn can guide users to make healthier choices and prevent diseases [9]. There is promising evidence on the effectiveness of mHealth interventions in the management of DM2 [10,11]. However, the evidence on the effect of mHealth interventions in prevention of DM2 is sparser. In a recently published systematic review it is emphasized that there is a need for further research on the effectiveness of mHealth interventions, particularly within people at risk of DM2 [10]. Only 1 [12] of 25 included studies were conducted on people at risk of DM2 [10].

Initiation and adherence to behavioral change is a complex process. In a previous randomized controlled trial (RCT), we found that long-term follow-up using an app to promote adherence to healthy behavior postcardiac rehabilitation (post-CR) was effective with regard to exercise capacity, exercise performance, exercise habits, and in self-perceived goal achievement [13]. Additionally, a 1.6 kilo difference were found between the groups, in favor of the intervention group, in bodyweight at 1 year follow-up [13]. Although this study was conducted on patients with cardiac diseases, the result is relevant as every kilogram of weight loss has shown a 16% risk reduction of DM2 incidence in people at risk of DM2 [14].

To be able to successfully initiate and adhere to behavioral change by using an app, it is necessary to consider the participants´ motivation for behavioral change as well as the participants’ motivation for using an app as an intervention (or as guidance) in the behavioral change process. In a previous qualitative study on post-CR patient´s experiences of using an app, we found that being followed by a real person and providing individualized feedback, most likely is the most significant success factor in promoting adherence to healthy behavior with an app [15]. Additionally, follow-up based on own goals was highlighted as important to increase motivation for both adherence to behavioral change and for using an app for this purpose [15].

Importantly, but not surprisingly, the relationship between health personnel and the patients with DM2 have been shown to influence clinical outcomes [16]. Despite this, most studies evaluating the effect of mHealth interventions in patients with DM2 are fully automated and are primarily self-managed [10,11]. This also seems to apply for people at risk of DM2 [12]. To our knowledge, no studies have developed and evaluated the effect of individualized follow-up incorporated in an app aiming to promote healthy behavior in people at risk of DM2.

Based on the described experiences and the current existing knowledge base, more knowledge on efficacy of mHealth interventions in prevention of DM2 is needed. Hence, we developed the People Living Under change (Plunde) app, aiming to promote initiation of behavioral change and adherence to healthy behavior. Before commencing a full scale RCT evaluating the effect of this app on risk reduction in people at risk of DM2, we plan to conduct a small scale RCT feasibility study to evaluate whether the full scale RCT can be conducted in the way it is planned or whether it needs to be modified. However, as our experiences with using apps primarily is in patients with cardiac diseases and the fact that experiences and perspectives of the end users need to be implemented in the development and evaluation of apps [17], testing the usability of the app in the target population ahead of conducting the feasibility study is warranted. Therefore, the aim of this study was to assess the usability of Plunde in people at risk of DM2.

## Methods

### Study Design, Setting, and Participants

A qualitative study with the use of a think aloud (TA) protocol [18] was conducted as described below. This study took place in the eastern part of Norway. Participants were recruited from Healthy life centers. Healthy life centers are a primary health care service implemented in about half of the municipalities in Norway, and aims to promote beneficial physical activity, diet, and tobacco behaviors [19]. Eligible participants were women and men over the age of 18 years having prediabetes or being at risk of developing DM2. They had to be familiar with and have some knowledge using smartphones and be able to read and understand Norwegian. Descriptive data included sex, age, education level, and level of familiarization with smartphone and apps.

### Theoretical Framework

Using an app as an intervention, or as a part of an intervention, can be considered as a complex intervention defined as an intervention containing several interacting components [20]. In order to be able to understand any change in lifestyle or effect of a complex intervention, a clear theoretical framework is known to be crucial [20,21]. In particular, applying a theoretical framework is associated with an increased likelihood of success in technology-based interventions [22]. Based on our previous research, it was important that Plunde contained the functions that the patients found to be crucial [15], which in turn are in line with the transtheoretical model of behavior change, also known as the stages of change model [23]. According to this model, change in health behavior involves 6 stages of change, and takes into account that changing a lifestyle is not a linear process [23]. These 6 stages include precontemplation stage, contemplation, preparation, action, maintenance, and termination. In the precontemplation stage, people do not intend to change their behavior for the next 6 months, while in the contemplation stage, people are aware of the pros of changing behavior. In the preparation stage, people intend to change behavior within the next month. People in the action stage have made specific modifications in their lifestyle. In the maintenance stage, the focus is on preventing relapse, and in the termination stage people are sure they will never return to their old, unhealthy behavior [23]. The need for support may be different from person to person as well as at different stages and should therefore be individualized in order to increase the likelihood of successful behavior change [23]. A relevant example is people at risk of DM2 which may be in the contemplation stage after getting information about their risk from their general practitioner and thereafter proceeds gradually to the maintenance stage. While the person at risk of DM2 in the contemplation stage needs support and advice related to planning and implementation of changes that are relevant and achievable, he or she may need less advice and more specific motivational feedback based on their actual new lifestyle and help for possible adjustments to promote adherence in the long term in the maintenance stage. To deal with the complexity of behavior change, the transtheoretical model uses different behavior strategies and techniques [23] which we carefully have tried to incorporate in Plunde.

### App Development and Main Features and Functions in Plunde

Based on previous research [10,11,24] and experiences with the use of an app to promote adherence to healthy behavior in post-CR patients [13,15,25], member of the research group (PL and BBN) created and drafted a prototype of the Plunde app, in cooperation with digital engineers at Simula Metropolitan Center for Digital Engineering (Simula Met). The very first version of the prototype was initially tested by members of the research group (PL, GS, GH, and BBN). In order to gain insight to the dimensions of lifestyle change in the target population (risk of DM2), a meta-synthesis of qualitative studies exploring facilitators and barriers for lifestyle change in people with prediabetes was conducted [26]. Based on the initial testing of the prototype and the meta-synthesis, minor adjustments of Plunde were made before the TA-sessions.

**Figure 1:**
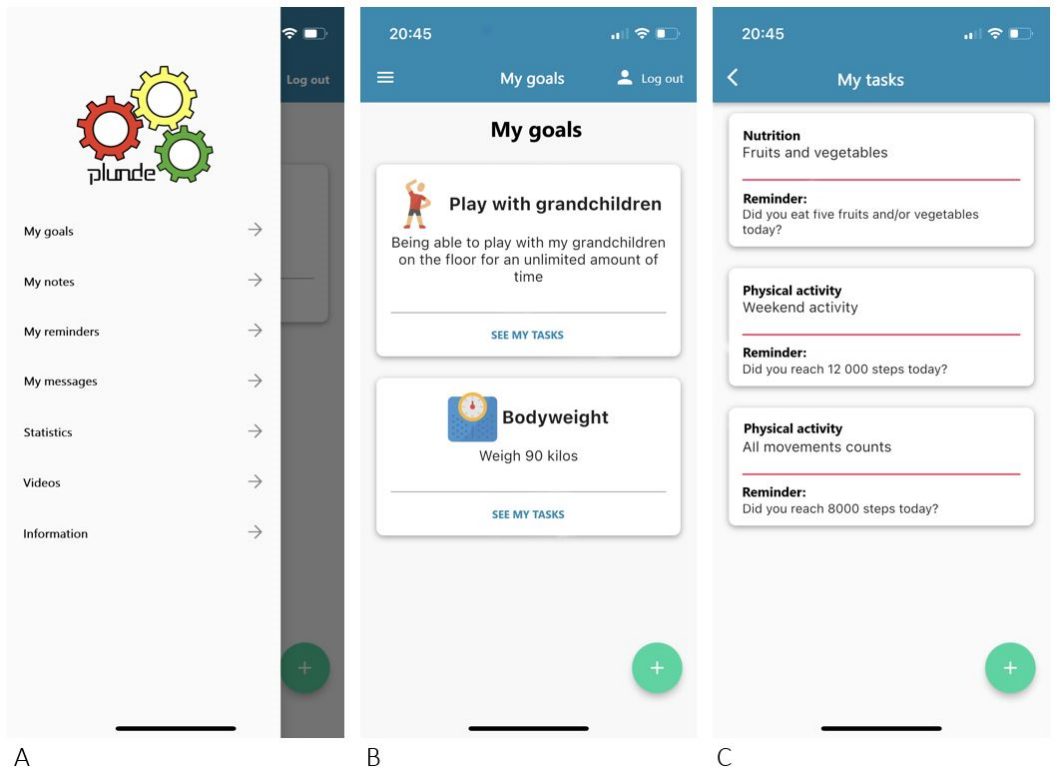
User interface of Plunde. (A) Menu with an overview of the features. (B) My goals. (C) Tasks related to the goals.

Using specific behavior change strategies and techniques in different stages of change may be useful in providing support, and thereby promoting adherence to healthy behavior [27]. An overview of the main features in Plunde are presented in Figure 1A. Goal-setting is considered to be an excellent method of promoting adherence [28] and was also highlighted in our previous research to increase motivation [15]. Therefore, individual goal setting was set as a prerequisite for using Plunde (Figure 1B). To each individual goal the user must decide tasks (Figure 1C) that should be done to reach the goal. Further, each task has an accompanying reminder. When and how often reminders of a task should appear, is decided by the user.

In addition to goal setting, a crucial function highlighted by patients in our previous research was individualized feedback provided by a real person [15]. Therefore, supervisors can monitor the participants using Plunde through an administrator interface. For the participants, they can receive individualized feedback from a supervisor (a health professional) through a message function. In this message function, the user can send messages to the supervisor as well. This function is considered as central as mHealth interventions providing individualized feedback has been proposed as a superior technique for long-term success [28]. Further, Plunde consists of relevant information which can be tailored to each user. This function was included as access to reliable health information can contribute to increased health literacy, which in turn can improve health [29,30]. In this context, participants can be informed on factors that influence the development of DM2 and ways to address these risk factors.

In addition to the mentioned functions, Plunde also includes a personal note function. This function is intended to support in self-monitoring which is found to be a motivational factor in changing behavior [31]. Users of Plunde can use this function as an exercise diary, for self-reflection regarding goal achievement in example, for logging their bodyweight, or for similar. These notes can be read by the supervisor in the administrator interface as well, which makes it possible to tailor the feedback to each participant even more.

### TA Procedure

The TA method enabled us to identify usability issues related to Plunde via observation and self-report [18]. The TA-session started with an observation of a predefined sequence of problem-solving tasks and involved asking the subjects to TA while solving the tasks (Textbox 1). This allowed us to observe the immediate reactions of the participants during the use of Plunde. A subsequent postactivity interview served to gain more in-depth knowledge of the users’ experience of the problem-solving tasks [32,33]. The problems-solving tasks (Textbox 1) and the interview guide (Multimedia Appendix 1) was developed and piloted prior to the TA-sessions. Before the TA-sessions, descriptive data were collected.

The problem-solving tasks was based on real-life scenarios addressing usability testing of all the main functions of Plunde. Based on experiences from previous research conducted by the research group [13,25], the flow of the problems-solving tasks was decided. The tasks were designed by 3 members of the research team (PL, GS, and BBN). The series of tasks were always conducted in a fixed order across the TA-sessions.

**Problem-solving tasks.**

- Go to the main menu and find “my goals”
- Find the tasks related to your goals
- Send a message to your supervisor
- Delete a message
- Create a note
- Delete the note
- Find the reminders that are linked to the tasks
- Change a reminder that is linked to one of the tasks
- Find information about healthy diet
- Find a video about physical activity

The TA-sessions were conducted in the period from April 2022 to November 2022. They took place at the localities of the different Healthy Life centers, at the University campus, or in the home or workplace of the participants. Participants were given a test phone being either an iOS or Android, depending on which operative system they had on their own smartphone. Each session started with a short introduction of Plunde and an explanation of the aim of the research project. Further, the procedure for testing the usability of Plunde was explained. The participants were instructed to TA (verbalization of thoughts) while performing the problem-solving activities and it was emphasized that the purpose of these tasks was not to measure their digital skills but to test the usability of Plunde.

The TA-sessions were recorded. Further, field notes were taken during the problem-solving tasks to record any observed technical difficulties encountered, ease of use, and learning as well as nonverbal behaviors related to the task management. The observer or interviewer (GS) reminded the participants to continue thinking aloud when they stopped doing so. If a participant was not able to solve a task after several attempts, the observer or interviewer provided a cue, in order to see whether and in what way, the task was solvable. After completing the TA-session, the participants received a cinema gift card valued 400 Norwegian kroner (US $40).

### Analysis and Material

The TA-sessions and the postactivity interviews were transcribed verbatim, and the data were coded independently by 2 members of the research group (GS and CFO). The principles of thematic analysis were followed and descriptive codes were developed [34]. The codes were compared and reviewed and then organized in preliminary and final themes emphasizing usability and usability issues. During this phase, it was decided whether the participants experienced the problem-solving tasks as difficult or not (Table 1). The decision was made on the basis of an overall evaluation of the participants´ answers in the interview, time spent on the tasks and number of cues given.

**Table 1:** Summary of results, usability issues related to tasks.

Task		TA^a^ participant number						
		TA1	TA2	TA3	TA4	TA5	TA6	TA7
1	Go to the main menu and find “personal goals”	–^b^	–	–	–	–	–	–
2	Find your personal tasks related to your goal(s)	–	–	–	–	–	–	–
3	Send a message to your supervisor	+^c^	–	–	–	–	–	+
4	Delete a message	+	+	+	–	+	+	+
5	Create a note	–	–	–	–	–	–	+
6	Delete the note	+	–	+	–	–	–	+
7	Find the reminders that are linked to the tasks	+	+	+	+	+	+	+
8	Change a reminder that is linked to one of the tasks	+	+	+	+	+	+	+
9	Find information about healthy diet	–	–	–	–	–	–	–
10	Find a video about physical activity	–	–	–	–	–	–	+

^a^TA: think aloud.

^b^–: no problems performing the task.

^c^+: problems performing the task.

### Ethical Considerations

Ethical approval was obtained from the Norwegian Centre of Research Data (ID: 887029). All included participants provided written informed consent.

## Results

### Participants and Their Characteristics

In total, 7 people at risk of DM2 with a median age of 66 (range 41-75) years participated in this study, 3 were women and 4 were men. Regarding educational level, 1 had finished primary education, 2 had finished high school, 3 had 1-3 years of college or university, and 1 had more than 3 years of higher education. In terms of smartphone and app use, 6 participants reported that they used apps every day and 1 participant reported several times per week. The participants responded to the statement “I have good skills and competence in the use of smartphones and applications” as follows; “highly agree” (n=2), “agree” (n=4), and “I both agree and disagree” (n=1).

### Usability

#### General Findings

On average, the TA-sessions lasted for a range of 44-67 (SD 8.3) minutes. The usability based on tasks completed in the TA observation are summarized in Table 1. The TA-sessions and the postactivity interviews evolved in three themes or topics: (1) user interface design of the app, (2) navigation strategy and functionality, and (3) suggestions for improvements to the functionality of the app.

#### User Interface Design

Feedback concerning the design of Plunde was mostly positive. It was pointed out by three of the participants that the layout was recognizable and similar to other apps, which made it easier to navigate based on experience. Most of the participants experienced that the menu was comprehensible. The participants had no problem finding the menu in Plunde. They liked that the menu was not overloaded with too much information. Additionally, most of the participants pointed that they liked the size of the font which made readability good. Generally, participants managed moving back and forth between the menu and different features, with few exceptions as mentioned below.

All participants located the personal goal feature and the tasks related to the personal goal. A novel feature of Plunde is the message feature that enables communication between participants with a supervisor (health care professional). Further, 2 participants found it challenging sending a message to the supervisor. However, this was solved quickly with cues from the interviewer. In order to delete a message in Plunde, the participant is required to swipe left. All except 1 participant had problems deleting a message once it was written. The participants kept searching for a button icon for deleting instead of swiping leftwards. When a cue was given, this was understood, but all the participants commented that this was illogical.

When continuing the navigation to the note feature, only one of the participants had some difficulties writing up a note. This participant could not find the keyboard at first. When given a cue about this, the participant continued only to lose the keyboard again. Further, 1 participant experienced some confusion regarding writing the headline of the note versus the content of the note as well as saving the note. Furthermore, deleting the note was perceived as easier than deleting a received message (problem-solving task number 4, Table 1). Despite this, 3 of the participants needed cues to accomplish the task of deleting the note. The delete commands differed between the message function and the note function, no double-confirmation was needed to delete a note. This was commented on by some of the participants.

When moving to the reminder part of the navigational task (problem-solving task numbers 7 and 8, Table 1), the participants were asked to set the day and time for an accompanying reminder to a personal task. All the participants encountered difficulties in locating and changing the reminders. Some needed cues related to the swiping function that enabled change. Others managed to change the date and time themselves. However, none of the participants managed to save the changes without cues from the interviewer. This was experienced as a usability issue.

None of the participants had trouble with the 2 tasks of finding information and videos related to lifestyle advice. Likewise, all but one found the link to a governmental health information video on physical activity. Further, 3 participants had trouble navigating back to Plunde from the video link. When cued by the interviewer, they managed this promptly.

#### Suggestions for Improvements to the Functionality of the App

All the participants pointed out some flaws in the design of Plunde that could be improved. The functions related to deleting and saving, both notes, tasks, and reminders, in Plunde were the most challenging. Hence most suggestions for improvement concerned these functions.

The delete function for messages was suggested to be changed from swiping to a “button/icon” to be tapped or double-tapped. A trashcan icon was suggested as an alternative as it was perceived as more in line with other apps familiar to the participants. Further, it was suggested that the save button for the personal notes feature should have been placed at the top of the page. What is more, to change the timing of reminders, a keyboard was suggested as a more user-friendly input method than the scroll function as in the current design of Plunde. Further, a snooze function for the reminders was requested by some of the participants.

Other improvement suggestions were related to the wording or labelling of the features and functions in Plunde. Further, 1 participant suggested that the command labelled “change” in the personal notes feature would be more understandable if labelled “change text.” Another participant suggested renaming the feature “information” which relates to evidence-based knowledge and guidelines regarding lifestyle. The label “information” was perceived to wrongfully be understood as information about Plunde*.* The participant suggested changing the label to “inspiration.”

Lastly, to aid in the navigation of Plunde, several of the participants suggested a help feature or alternatively a separate written manual. In this connection, 1 participant expressed that an app should be so intuitive that there would be no need for a help feature.

## Discussion

### Principal Findings

The aim of this study was to assess the usability of an app developed to promote initiation of behavioral change and adherence to healthy behavior in people at risk of DM2. In general, only minor changes to the functionality of Plunde was reported as necessary to improve the usability. The most critical improvement included how to delete a message as this was difficult for all except one. This function is considered as crucial since it is important to maintain control over your own data. The participants are told not to share any sensitive information; however, it may also happen that they share something else that they later on would like to delete for some reason. To make the design consistent, deleting a message and deleting a note has therefore been changed so these 2 commands are the same (swiping right). Additionally, a trashcan symbol has been added in addition to the text “delete.” Other improvements made based on the TA-sessions included how to save notes and renaming of the feature “information.” As suggested by some of the participants, the command saving has been moved to a more obvious place (right top of the interface) and a symbol for saving has been added as well. The feature “information” was suggested to be changed to “inspiration.” Since this word does not cover the intended content, the new label “knowledge assembly” was landed on after a discussion in the research group. To further improve the usability by making the design more appealing, small changes such as colors and icons have been made.

Based on the results, finding and changing reminders that are linked to the tasks also should be considered to be changed. However, as Plunde will be used in research where it is not desirable for participants to change goals, tasks, or reminders throughout an intervention period, we have chosen not to change this function at this time. Before implementation of Plunde to clinical practice, this should be considered to increase the usability. Some of the findings from this TA study were not relevant to the upcoming small scale RCT feasibility study but might have to be considered in potential upcoming studies as well as for the implementation of Plunde.

### Strengths and Limitations

The sample of this study may seem small. However, previous studies have established that 80%-90% of the usability issues of web sites and apps can be detected in samples of 5 to 9 participants [35,36]. Throughout the individual TA-sessions the themes were repeated and there was an understanding that data saturation was reached [37]. However, we cannot exclude that different usability issues and more variation of perspectives might have arisen by including more participants. Additionally, most of the participants were older than 60 years and no younger than 40 years. We would have expected different results in a sample of younger people or people with different demographical backgrounds [38,39]. As the sample in this study consisted mainly of elderly people, it is important to consider the age associated changes in terms of usability [40]. Studies comparing young and older adults’ use of smartphones conclude that there are 5 distinct human factors where older adults are different from younger people: learning time, speed of performance, error rate, retention over time, and subjective satisfaction [38]. It is therefore important to strive for as representative a sample as possible in the planned feasibility study.

The TA method has been criticized because of the high degree of self-reported data, which may jeopardize the validity of this study [41]. To increase the objectivity of the data we could have used video recording and eye-tracking. However, this method demands more time and resources and generates a large amount of data. Therefore, we chose a more pragmatic approach. A strength of this study is the use of the same facilitator or interviewer through all the TA-sessions. This was an important move to strengthen the validity of the results since it helped to standardize the TA-session process in terms of when to cutoff or intervene as well as encourage them to look to the facilitator for help in completing the tasks early [42]. That being said, the provision of cues in the usability testing could have been done in a more systematic way by counting the number of attempts and number of cues needed to solve a task. This varied in the interviews and could potentially affect the credibility of the findings. Further, it should be considered that the TA method is conducted in a constructed setting and is not necessarily comparable to real life settings [43]. It is important to consider that many people use their smartphones when they are “on the go” being surrounded by noise, other people, and traffic. However, 1 important strength of the TA method is that the immediate reactions and thoughts when using Plunde is captured [42]. Many people, perhaps especially older people, might have difficulty in recalling usability issues if interviewed after some time.

### Future Research

It remains an open question how quickly test persons would adapt to the design and features of Plunde and how satisfied they would be when using it over a longer period of time. These questions are intended to be answered by an upcoming feasibility RCT where the aim is to investigate how a future full scale RCT can be conducted. For the feasibility study, we plan to include 60 participants into three study arms: (1) Plunde app, (2) lifestyle intervention at a Healthy Life Centre (usual care), or (3) Plunde app + lifestyle intervention at a Healthy Life Centre. The primary outcome in this study is feasibility and criteria for success will be preset. If Plunde turns out to be feasible, we will consider testing it further for effect on bodyweight (primary outcome) in a full-scale RCT in people at risk of DM2.

### Conclusions

Feedback from the participants gained through the TA-sessions and the postactivity interviews indicated their preferences for Plunde. In general, participants were satisfied with the usability of Plunde. By asking participants to navigate through, and comment on the features in Plunde as described in the problem-solving tasks, the researcher gained real time perspective on the appeal, relevance, and utility of Plunde. This has been translated into a refined version of Plunde that will be used in a feasibility study as described.

## Appendix

Multimedia Appendix 1 Interview guide, post-activity interview.

## Abbreviations

DM2: type 2 diabetes mellitus
mHealth: mobile health
Plunde: People Living Under change
Post-CR: postcardiac rehabilitation
RCT: randomized controlled trial
TA: think aloud
