# Supplementary material for: Think Aloud Testing of a Smartphone App for Lifestyle Change Among Persons at Risk of Type 2 Diabetes: Usability Study
Source: JMIR Hum Factors. 2023 Nov 15;10:e48950. doi: 10.2196/48950 (PMC10687681; doi:10.2196/48950)
Supplement: Multimedia Appendix 1 [file humanfactors_v10i1e48950_app1.docx]

**Interview guide**

How do you think this went?

Was it something you found easy?

Was there anything you found particularly difficult?

You mentioned something about x. Can you say a bit more about it, please?

Do you have any suggestions for improvement?

Can you imagine that this is an app you would have used in your everyday life, possibly why/why not?

Do you have anything else you would like to add?

Do you have any questions before we are finished?
